# Structure of the O‐Antigen and the Lipid A from the Lipopolysaccharide of *Fusobacterium nucleatum* ATCC 51191

**DOI:** 10.1002/cbic.202000751

**Published:** 2020-12-14

**Authors:** Pilar Garcia‐Vello, Flaviana Di Lorenzo, Dimitra Lamprinaki, Anna Notaro, Immacolata Speciale, Antonio Molinaro, Nathalie Juge, Cristina De Castro

**Affiliations:** ^1^ Departmentof Chemical Sciences University of Naples Federico II Via Cinthia, 26 80126 Napoli NA Italy; ^2^ Gut Microbes & Health Institute Strategic Programme Quadram Institute Bioscience Rosalind Franklin Road, Norwich Research Park Norwich NR4 7UQ UK; ^3^ Department of Agricultural Sciences University of Naples Federico II Via Università, 100 80055 Portici NA Italy

**Keywords:** *Fusobacterium nucleatum*, Gram-negative bacteria, lipid A, lipopolysaccharides (LPS), MALDI-TOF mass spectrometry, NMR spectroscopy, O-antigens

## Abstract

*Fusobacterium nucleatum* is a common member of the oral microbiota. However, this symbiont has been found to play an active role in disease development. As a Gram‐negative bacterium, *F. nucleatum* has a protective outer membrane layer whose external leaflet is mainly composed of lipopolysaccharides (LPSs). LPSs play a crucial role in the interaction between bacteria and the host immune system. Here, we characterised the structure of the O‐antigen and lipid A from *F. nucleatum* ssp. *animalis* ATCC 51191 by using a combination of GC‐MS, MALDI and NMR techniques. The results revealed a novel repeat of the O‐antigen structure of the LPS, [→4)‐β‐d‐Glc*p*NAcA‐(1→4)‐β‐d‐Glc*p*NAc3NAlaA‐(1→3)‐α‐d‐Fuc*p*NAc4NR‐(1→], (R=acetylated 60 %), and a bis‐phosphorylated hexa‐acylated lipid A moiety. Taken together these data showed that *F. nucleatum* ATCC 51191 has a distinct LPS which might differentially influence recognition by immune cells.

## Graphical abstract

**Strain‐specific features**: Characterization of the lipopolysaccharide from *F. nucleatum* ssp. *animalis* ATCC 51191 revealed a novel O‐antigen structure for bacterial LPS [→4)‐β‐d‐GlcpNAcA‐(1→4)‐β‐d‐GlcpNAc3NAlaA‐(1→3)‐α‐d‐FucpNAc4NR‐(1→], and a bis‐phosphorylated hexa‐acylated lipid A moiety. These data showed that *F. nucleatum* ATCC 51191 has a novel O‐antigen, and pave the way towards understanding the immunological perception of this macromolecule.

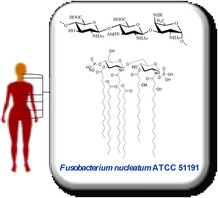

## Introduction

*Fusobacterium nucleatum* is an anaerobic Gram‐negative bacterium and a component of the human oral microbiota.^[1]^ Within the oral cavity *F. nucleatum* adhesins can also interact with microbes and host cells for plaque biofilm development.^[2]^ However, several studies have highlighted a plausible switch of *F. nucleatum* from being a commensal to act as a pathosymbiont as some adverse health outcomes have been associated to its persistence within the human body.^[3]^ Despite the identification of a range of virulence factors, the molecular mechanisms by which *F. nucleatum* contributes to these non‐oral diseases remain unclear.[^4^, ^5^]

As a Gram‐negative bacterium, *F. nucleatum* is surrounded by an outer membrane covering the thin peptidoglycan cell wall, protecting the bacteria from hostile environments.^[6]^ The external face of the outer membrane is composed mainly by lipopolysaccharides (LPS).^[6]^ The LPS molecule is typically divided into three different moieties: lipid A, core oligosaccharide and O‐antigen. Lipid A is a phosphoglycolipid typically composed by a glucosamine disaccharide, phosphate groups and acyl chains; it is the hydrophobic part of the LPS and serves as an anchor into the outer membrane. Lipid A has covalently linked an oligosaccharidic part called the core region. The latter is composed of 3‐deoxy‐d‐*manno*‐oct‐2‐ulosonic acid (Kdo) and l‐*glycero*‐d‐*manno*‐heptose residues in addition to other more common monosaccharides. Finally, the O‐antigen, is made of several repeating units that can be composed by up to nine monosaccharides.[^7^, ^8^]

As a microbe associated molecular pattern (MAMP), LPS plays a crucial role in the bacteria‐host interactions as it is recognized by host pattern recognition receptors (PRRs), resulting in activation of the immune response. This is mainly mediated by the innate immune TLR4/MD‐2 complex dimerization upon recognition of the LPS lipid A, which drives the production of pro‐inflammatory cytokines.[^9^, ^10^] This recognition and the following signalling cascade is strictly dependent on the structure of the lipid A.[^9^, ^10^] In this context, it was shown that *F. nucleatum* LPS can stimulate B lymphocytes^[11]^ and induce expression of tumour necrosis factor alpha (TNF‐α) and interleukin 8.^[12]^

However, the dual commensal‐pathogen behaviour of *F. nucleatum* highlights the importance to determine in detail the structure of its LPS. In this context, the chemical structure of the LPS O‐repeating units of *F. nucleatum* strains ATCC 23726,^[13]^ MJR 7757B,^[14]^ 10953,[^6^, ^15^] 12230^[16]^ and 25586^[17]^ have been characterised. Also, the composition of the LPS from *F. nucleatum* strain JCM 8532^[18]^ and the composition of the LPS and the lipid A of *F. nucleatum* ATCC 25586 (Fev1)[^19^, ^20^] have been reported. These data showed high diversity in carbohydrate composition among strains, whereas the presence of amino sugars, uronic acids and amino acetylating groups is a common feature of these strains.

Here we report the structural characterisation of *F. nucleatum* spp. *animalis* ATCC 51191 LPS focusing on the O‐antigen and lipid A as determined by gas liquid chromatography–mass spectrometry (GLC‐MS), matrix‐assisted laser desorption/ionization (MALDI) mass spectrometry (MS) and NMR spectroscopy.

## Results

### LPS extraction, purification and compositional analysis

LPS from *F. nucleatum* spp. *animalis* ATCC 51191 cells was isolated by hot water/phenol extraction.^[21]^ LPS was further purified by enzymatic treatment, centrifugation and ultracentrifugation steps. SDS‐PAGE analysis of the water layer of the extract showed the presence of two main groups of bands, suggesting that this bacterium produces smooth (S)‐type LPS (LPS with O‐polysaccharide) and rough (R)‐type LPS (composed only of lipid A and core region). The apparent molecular weight of the S‐type LPS was estimated between 25 and 35 kDa (Figure S1 in the Supporting Information). The monosaccharide composition of purified LPS was analysed by derivatization to acetylated *O*‐methyl glycosides and revealed the presence of glucose (Glc), heptose (probably l‐*glycero*‐d‐*manno*‐heptose, based on the similar chromatographic retention time), and traces of Kdo; no other monosaccharides could be detected using this approach, probably due to the resistance to cleavage of the aminuronic acids composing the O‐antigen. These units, along with the 2,4‐diamino‐2,4,6‐trideoxydeoxy‐galactose (Fuc*p*N4N) were detected during the NMR analysis. The fatty acid analysis revealed the presence of tetradecanoic acid (14 : 0), 3‐hydroxytetradecanoic acid (14 : 0 3‐OH), hexadecanoic acid (16 : 0) and 3‐hydroxyhexadecanoic acid (16 : 0 (3‐OH); Figure S2b).

**Figure 1:**
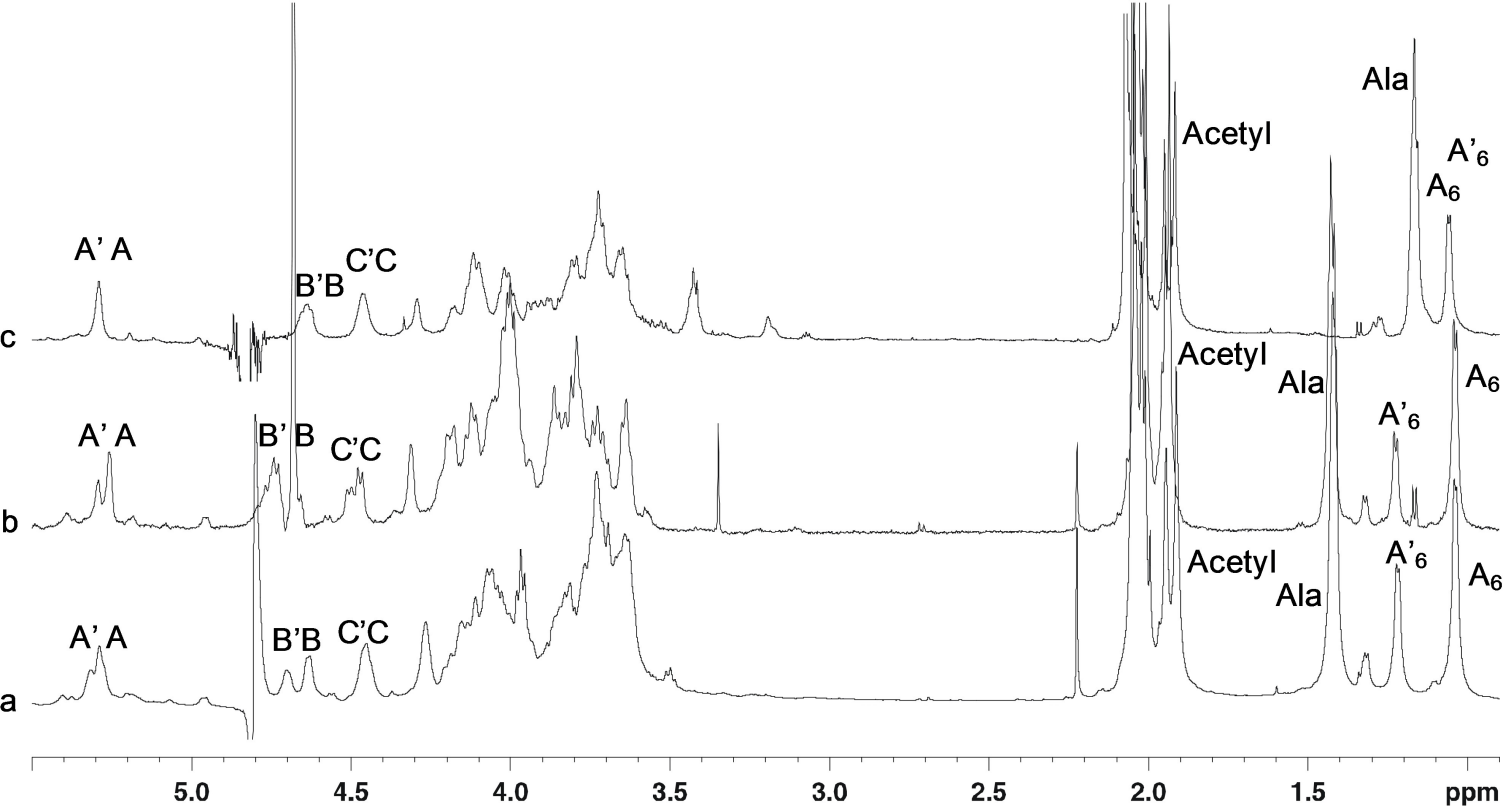
^1^H NMR spectrum (600 MHz) of the O‐antigen of *F. nucleatum* ATCC 51191. The analysis was carried out at a) neutral pH (550 μL D_2_O, 25 °C), b) acid pH (550 μL D_2_O+4 μL of DCl conc, 37 °C) and c) alkaline pH (550 μL D_2_O+4 μL of NaOD 4 M, 25 °C).

A mild acid hydrolysis of the purified LPS was then carried out in order to analyse separately the lipid A and the O‐antigen fraction. The lipid A, obtained as a precipitate after centrifugation of the acid hydrolysis product, was analysed via MALDI‐TOF MS and MS/MS, while the polysaccharide part was further purified by size exclusion chromatography (Figure S3). Among all fractions in the chromatographic profile (Figure S3), only one had the characteristic of a polysaccharide (yield 55.5 %) and was analysed by 1D and 2D NMR (Figure 1). The first fraction did not produce relevant signals in the proton NMR spectrum, which suggested that it could a minor fraction of LPS that survived to the mild acidic treatment. The material eluted after the O‐antigen, contained carbohydrate material with several anomeric signals with no apparent stoichiometric ratio, which suggested that is was a mixture of oligosaccharides deriving from the core region of the rough component of the LPS. This fraction was not investigated further.

### Structural characterization of the O‐antigen by NMR

The structure of the O‐antigen part of *F. nucleatum* ATCC 51191 LPS was determined by analysing homo‐ and heteronuclear 2D NMR experiments recorded by dissolving this glycan in D_2_O. ^1^H, ^1^H COSY and ^1^H, ^1^H TOCSY experiments were used to disclose the protons of each spin system; each carbon atom was identified through the analysis of the ^1^H, ^13^C HSQC. Finally, the primary sequence was inferred by analysis of *inter*‐residue and long‐range dipolar and scalar correlations from ^1^H, ^1^H NOESY and ^1^H, ^13^C HMBC spectra, respectively.

The ^1^H NMR spectrum (Figure 1) presented six main anomeric signals in the range 5.5–4.4 ppm and a crowded carbinolic region (4.3–3.4 ppm); furthermore, eight signals in the region of the methyl groups were detected (2.1–1.0 ppm). These were identified as the methyl groups of acetyl groups (2.1–1.8 ppm), of alanine (1.43 ppm) and of two 6‐deoxy sugars (1.23 and 1.04 ppm; Figure 1). In the HMBC spectrum, several signals were observed in the region of the carbonyl groups between 172–176 ppm, consistent with the presence of the alanine group, two uronic acid residues and several acetyl moieties.

**Figure 2:**
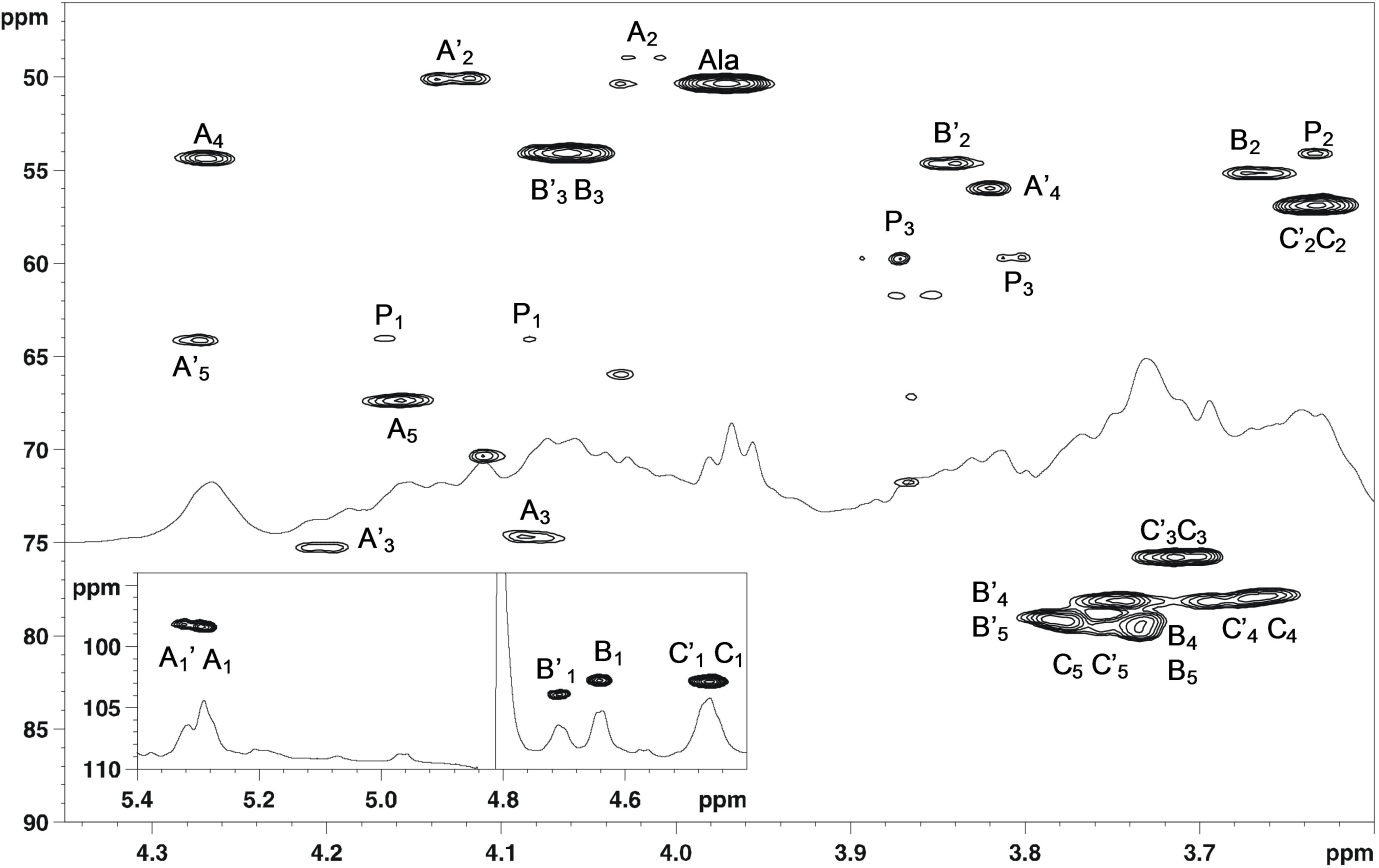
^1^H,^13^C HSQC spectrum of the O‐antigen of *F. nucleatum* ATCC 51191 (600 MHz, 25 °C, 550 μL of D_2_O, neutral pH). P1 and P3 densities are related to minor species, whose identity could not be established.

Three different monosaccharide residues were found to compose the *F. nucleatum* ATCC 51191 O‐antigen repeating unit: that is, β‐d‐Glc*p*NAcA, β‐d‐Glc*p*NAc3NAlaA and α‐d‐Fuc*p*NAc4NAc. However, the anomeric region of the spectrum presented six relevant signals indicative of non‐stoichiometric substitutions; the spin systems were labelled in couples as **A**–**A’**, **B**–**B’** and **C**–**C’** (Figures 1 and 2, Table 1).

**Table 1:** Proton (^1^H) and carbon (^13^C; *italic*) NMR chemical shifts of the O‐antigen of *F. nucleatum* ATCC 51191 (600 MHz, 25 °C, 550 μL of D_2_O, neutral pH). The chemical shifts of the methyl of the acetyl groups were at about ^1^H/^13^C 2.1‐1.8/23 ppm.

Residue	1	2	3	4	5	6
A’ →3)‐α‐d‐Fuc*p*NAc4N‐(1→	5.32	4.02	4.20	3.82	4.27	1.24
	*98.2*	*48.9*	*75.2*	*56.0*	*64.1*	*16.2*
A →3)‐α‐d‐Fuc*p*NAc4NAc‐(1→	5.29	4.13	4.09	4.27	4.17	1.04
	*98.4*	*50.1*	*74.6*	*54.3*	*67.3*	*16.5*
B’ →4)‐β‐d‐Glc*p*NAc3NAlaA‐(1→	4.71	3.84	4.08	3.78	3.78	
	*103.9*	*54.5*	*54.0*	*79.1*	*79.1*	*176.5*
B →4)‐β‐d‐GlcpNAc3NAlaA‐(1→	4.64	3.67	4.06	3.73	3.73	
	*102.8*	*55.2*	*54.0*	*79.3*	*79.3*	*176.5*
C’ →4)‐β‐d‐Glc*p*NAcA‐(1→	4.47	3.65	3.73	3.69*	3.75	
	*102.0*	*56.9*	*75.5*	78.1*	*78.0*	*176.5*
C →4)‐β‐d‐Glc*p*NAcA‐(1→	4.46	3.63	3.71	3.67*	3.75	
	*102.9*	*56.7*	*75.5*	77.9*	*78.0*	*176.5*
Ala	*– 172.6*	3.98 *50.2*	1.43 *18.1*

*C4 and C’4 attribution can be exchanged.

**Figure 3:**
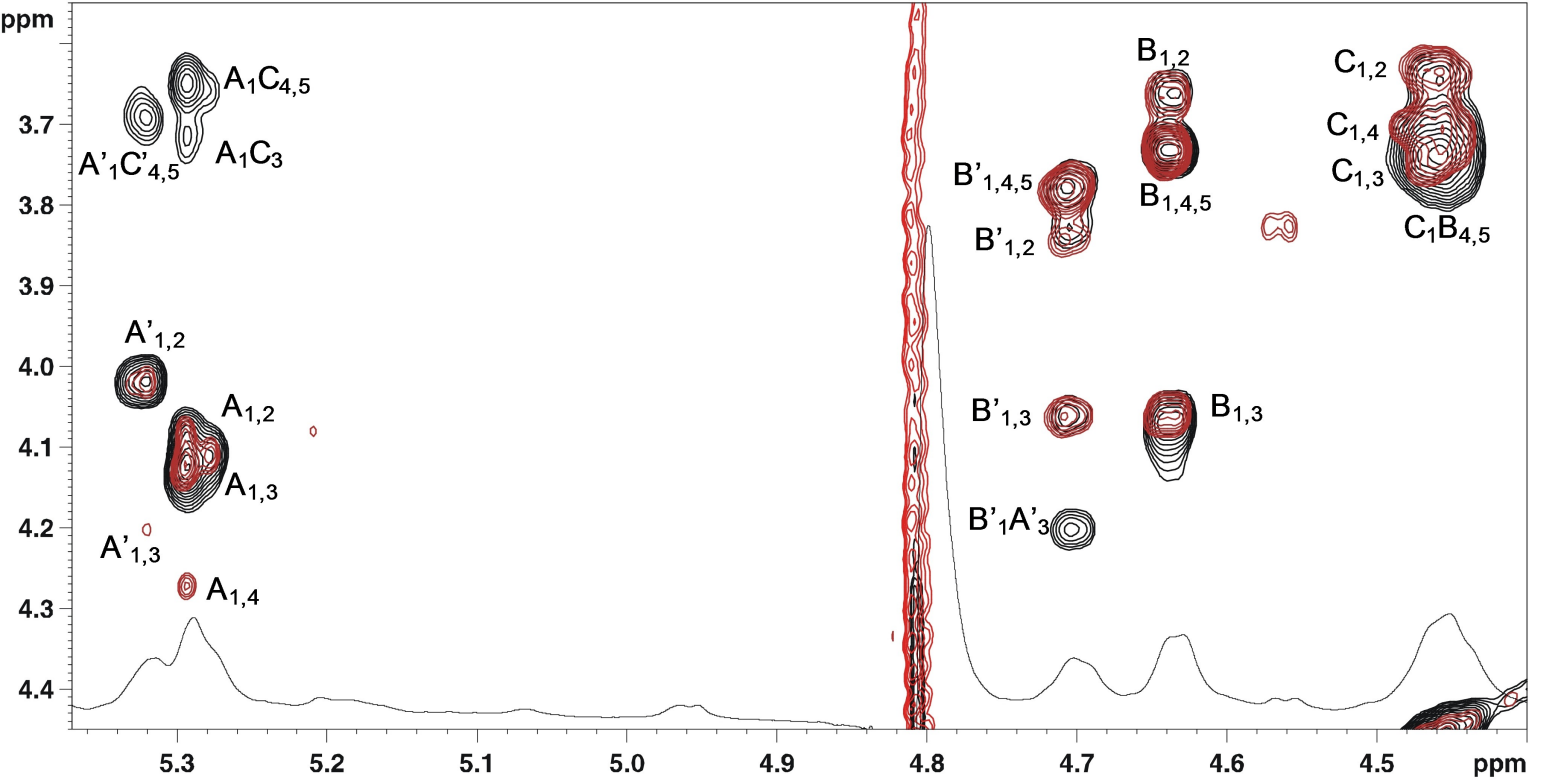
^1^H TOCSY (red) and ^1^H NOESY (black) spectra of the O‐antigen of *F. nucleatum* ATCC 51191 (600 MHz, 25 °C, 550 μL of D_2_O, neutral pH).

The NMR analysis of residue **A’**, whose anomeric proton (5.32 ppm) correlated with H‐2, H‐3 and H‐4 in the TOCSY spectrum (Figure 3), suggested a *galacto*‐configured residue. Combining the analysis of the TOCSY and COSY spectra with those of the HSQC spectrum, the corresponding carbon chemical shifts were identified (Table 1). Identification of the C‐4 value allowed assignment of the methyl group at 1.24 ppm, based on the corresponding long‐range correlation in the HMBC and also provided information about H‐5/C‐5 chemical shifts. Thus, **A’** was identified as a Fuc*p*N4N based on the diagnostic carbon chemical shifts of C‐2 and C‐4 (48.8 and 55.9 ppm, respectively) characteristic of nitrogen bearing carbon atoms. Based on the H‐2 and H‐4 chemical shifts, it was possible to establish that the amino group at C‐2 was acetylated (H‐2 at 4.02 ppm) while the C‐4 was not (H‐4 at 3.82 ppm). The α configuration of the anomeric centre was inferred by the chemical shift of the anomeric proton, by the presence of a NOE correlation between H‐1 and H‐2 of **A’**, and further confirmed by comparison with literature data.[^17^, ^22^] The analysis of residue **A**, conducted as above for **A’**, indicated that it also corresponded to an α‐d‐Fuc*p*2N4N. However, there was a significative difference in the proton chemical shifts between H‐4 of **A’** (3.82 ppm) and H‐4 of **A** (4.27 ppm), indicating that N‐4 of **A** was acetylated.

The analysis of the NOESY spectrum showed that the anomeric proton of **A** and **A’** had one correlation at about 3.73 ppm, an area of the spectrum which contained several proton signals, belonging to residues **C** (and **C’**) and to **B** (or **B’**; Figure 3). However, the linkage of **A’** to **B’** was ruled out because **B** was connected to **A** as inferred by the NOE effect between H‐1 of **B** with H‐3 of **A** (vide infra). For this reason, **A** (or **A’**) was connected to **C** (or **C’**). The exact point of substitution was confirmed in a further NMR analysis by varying the pH (as described below), which decreased the overlap between the signals in that region of the proton spectrum.

The anomeric proton **C’** (4.47 ppm) was almost coincident with that of **C** (4.46 ppm) at neutral pH (Figure 1a), while it appeared at a distinct chemical shift when the spectra were recorded at acidic pH (Figure 1b). For both **C** and **C’**, the correlation pattern in the TOCSY spectrum suggested a β‐*gluco*‐configured residue (Figure 3), with all the ring protons next to each other at 3.77–3.61 ppm. Identification of H‐2 protons was inferred by analysing the COSY spectrum, while identification of H‐3 and H‐5 protons was possible by observing the H‐1/H‐3 and H‐1/H‐5 NOE's effects, with the sorting between H‐3 and H‐5 driven from the correlation in the COSY spectrum, then the density left out in the TOCSY spectrum was assigned to H‐4.

The HSQC showed a chemical shift for C‐2 at 56.9 ppm evidencing the presence of an amino function, and the HMBC related H‐4 and H‐5 to a C‐6 at 176.5 ppm thus disclosing its nature as an uronic acid. Therefore, based on this information, **C’** and **C** were identified as two units of glucosaminuronic acid (Glc*p*NA). However, due to the crowded region of signals in the HSQC spectrum, the assignment of the C‐3, C‐4 and C‐5 values of this residue was confirmed by analysing the spectra recorded at acidic pH.

The analysis of the NOESY spectrum (Figure 3) was not resolutive, because **C’** and **C** had a very intense NOE in a region were their H‐3 and H‐5 appeared along with the protons of **B’** and **B**. The information that **C’** (or **C**) was linked to O‐4 of **B’** (or **B**) was inferred by analysing the spectra at acidic pH.

Residues **B** and **B’** were analysed in a similar way to **C** and identified these as a β‐*gluco* configured unit. However, **B** (or **B’**) presented amino functions on both C‐2 and C‐3, with N‐3 bearing an alanine as proved by the correlation in the HMBC spectrum between H‐3 and a carbonyl at 172.6 ppm.^[23]^ According to the Carbohydrate Structure Database (CSDB),[^24^, ^25^] GlcN3NA is present in different bacterial LPS and with the amino group at position C‐3 bearing other groups including L‐alanine (and never d‐) which most of the time is further decorated with a formyl group or with an acetyl. The lack of signal at about 8.0 ppm in the proton spectrum indicated that in the *F. nucleatum* ATCC 51191 O‐antigen the formyl moiety was not present (Figure S4).

Finally, residue **B’** was proposed to be linked to O‐3 of **A’** based on the NOE correlation between the anomeric proton signal of **B’** and H‐3 of **A’** (Figure 3); the same connectivity was assumed between **B** and **A** because the corresponding NOE was not visible since it overlapped with that between H‐1 of **B** and its H‐3.

**Figure 4:**
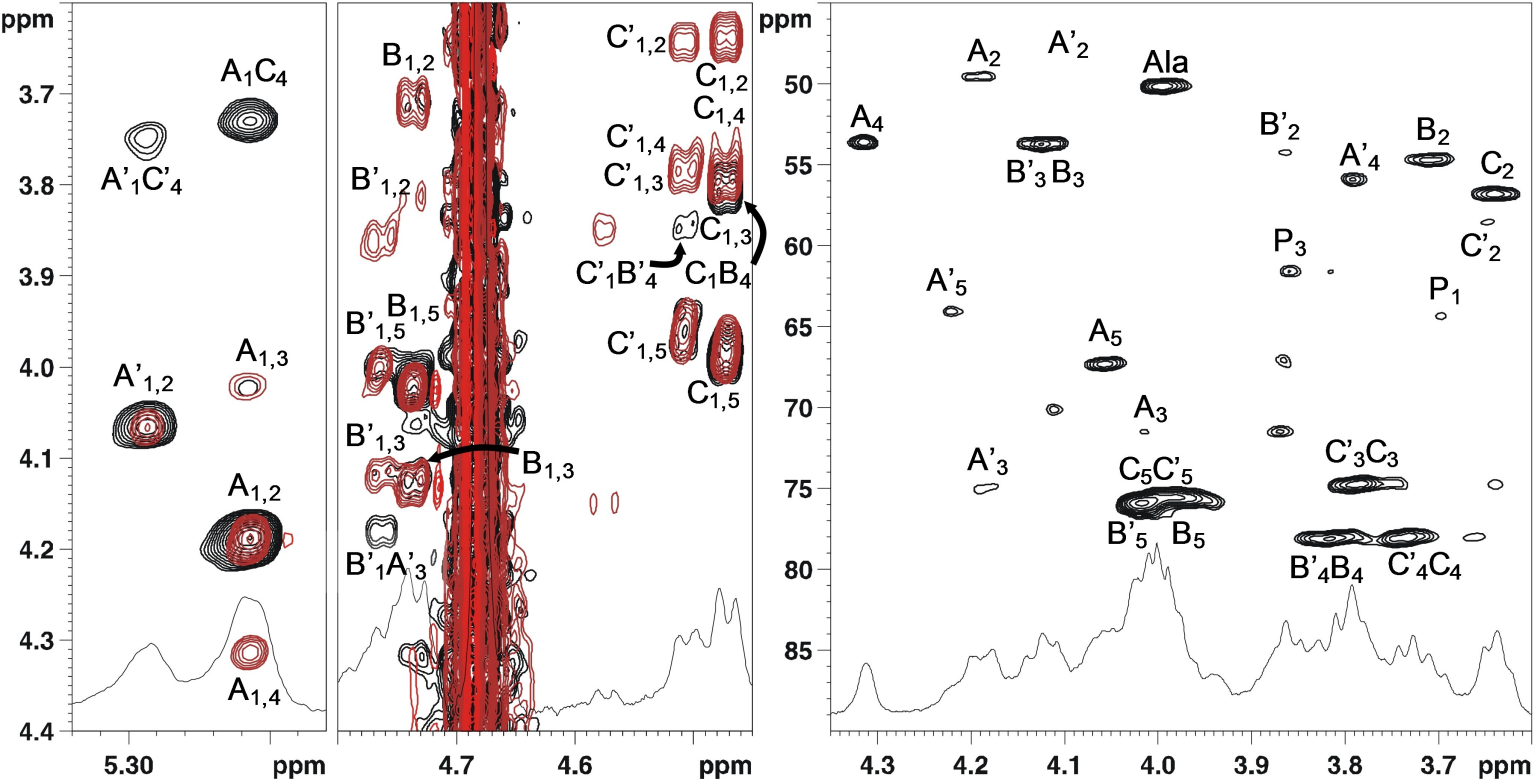
Left and middle: ^1^H TOCSY (red) and ^1^H NOESY (black) spectra at acid pH; right: ^1^H,^13^C HSQC of the O‐antigen of *F. nucleatum* ATCC 51191 (600 MHz, 37 °C, 550 μL D_2_O+4 μL of DCl conc, acid pH). P_1_ and P_3_ densities related to minor species, whose identity could not be established.

Together, the above NMR analysis identified two partial sequences: β‐d‐GlcNAc3NAlaA‐(1→3)‐α‐d‐FucNAc4 N (**B’**→**A’**), and β‐d‐GlcNAc3NAlaA‐(1→3)‐α‐d‐FucNAc4NAc (**B**→**A**), respectively. The absolute configuration for these three units was assumed to be d since this is the only isomer reported for each of them in the CSDB database.^[24]^ Similarly, the absolute configuration of Ala was assumed to be l, as the CSDB database reported only l‐Ala and never d‐Ala attached to GlcN3NA (last query placed on October 2020). However, at neutral pH, it was not possible to locate **C** (or **C’**) in the sequence due to the overlap occurring for some of the proton signals. In order to determine the full sequence of the O‐antigen repeating unit, NMR spectra were acquired at acidic and alkaline pH, with the one at acid pH being the most resolutive (Table 2). Following the same approach as above, the analysis of the NMR spectra led to the unequivocal identification of the C‐3, C‐4 and C‐5 of **C’** and **C** (Figure 4, Table 2) that, combined with the results from the NOESY spectrum, showed that **A’** (or **A**) was linked to O‐4 of **C’** (or **C**; Figure 4). The NOESY spectrum revealed the presence of a glycosidic bond between C‐1 of **C’** and the O‐4 of **B’**. The same connectivity was proposed for **C** and **B** as the corresponding NOE overlapped with that of the H‐3 of **C** and, therefore, was not visible (Figure 4). Moreover, the evident change of the H‐5 chemical shifts of **B** and **C** at acidic pH (**B**: 3.75 vs. 4.02 ppm; **C**: 3.67 *vs* 3.98 ppm), confirmed that the carboxylic groups (residues **B/C** and **B’/C’**) were free and not amidated.

**Table 2:** Proton (^1^H) and carbon (^13^C; *italic*) NMR chemical shifts of the O‐antigen of *F. nucleatum* ATCC 51191(600 MHz, 37 °C, 550 μL D_2_O+4 μL of DCl conc, acid pH).

Residue	1	2	3	4	5	6
A’	5.30	4.07	4.19	3.79	4.22	1.22
→3)‐α‐d‐Fuc*p*NAc4N‐(1→	*98.5*	*48.8*	*75.0*	*55.8*	*64.0*	*16.3*
A	5.26	4.19	4.01	4.32	4.06	1.04
→3)‐α‐d‐Fuc*p*NAc4NAc‐(1→	*98.8*	*49.5*	*71.5*	*53.6*	*67.3*	*16.7*
B’	4.74	3.87	4.13	3.82	4.02	
→4)‐β‐d‐Glc*p*NAc3NAlaA‐(1→	*102.4*	*54.2*	*53.7*	*75.9*	*75.9*	*176.1*
B	4.74	3.71	4.13	3.82	4.02	
→4)‐β‐d‐Glc*p*NAc3NAlaA‐(1→	*102.4*	*54.6*	*53.7*	*75.9*	*75.9*	*176.1*
C’	4.51	3.64	3.97	3.73	3.98	
→4)‐β‐d‐Glc*p*NAcA‐(1→	*102.3*	*56.7*	*75.5*	*77.9*	*78.0*	*176.1*
C	4.48	3.64	3.97	3.73	3.98	
→4)‐β‐d‐Glc*p*NAcA‐(1→	*102.5*	*56.7*	*75.5*	*77.9*	*78.0*	*176.1*
Ala		3.99	1.42			
	*172.6*	*50.1*	*18.0*

The sample solved in alkaline condition (Figure 1c) was not further investigated because of the strong overlap between the two sets of anomeric signals. However, the spectrum in alkaline condition reported a shift at high field for the methyl group of alanine, which suggested that the amino function of this aminoacid was not capped with any acyl, but present in the free form.

**Figure 5:**
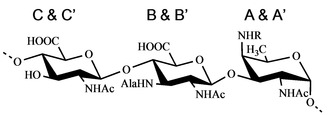
Structure of the O‐antigen isolated from *F. nucleatum* ATCC 51191. 4)‐β‐d‐Glc*p*NAcA‐(1→4)‐β‐d‐Glc*p*NAc3NAlaA‐(1→3)‐α‐d‐Fuc*p*NAc4NR‐(1→. R=acetyl (60 %). When R=Ac, residues are labelled **A**, **B** and **C**, when R=H residues are labelled **A’**, **B’** and **C’**.

In conclusion, the O‐Antigen repeating unit of *F. nucleatum* ATCC 51191 has been identified as [→4)‐β‐d‐Glc*p*NAcA‐(1→4)‐β‐d‐Glc*p*NAc3NAlaA‐(1→3)‐α‐d‐Fuc*p*NAc4NR‐(1→], R=Acetyl or H (Figure 5).

Hence, the amino function at C‐4 of the α‐d‐Fuc*p*2NAc,4N (**A**‐**4** and **A’**‐**4**) is acetylated in a non‐stoichiometric fashion, and the integration of the HSQC densities of **A** and **A’** revealed that acetylation occurs for approximately 60 % of the residues. The presence (or absence) of the acetyl units influences the chemical shifts of the other sugars, so that two different repeating units are identified by NMR.

### Structural characterization of the lipid A by MALDI‐TOF MS

**Figure 6:**
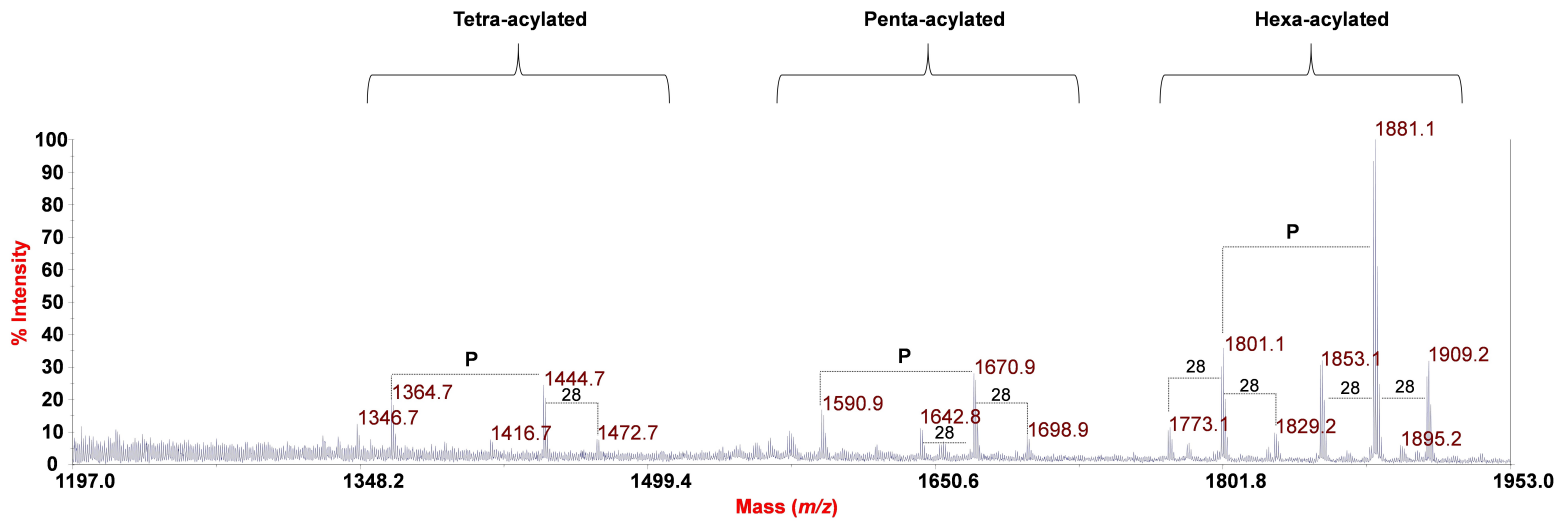
Negative‐ion MALDI‐TOF (reflectron mode) mass spectrum of the lipid A of *F. nucleatum* ATCC 51191. Differences of 28 amu are reported in the spectrum. The families of lipid A species differing in the acylation degree were also indicated as “hexa‐acylated”, “penta‐acylated” and “tetra‐acylated”. “P” indicates the phosphate group.

The negative‐ion MALDI‐TOF MS spectrum of the lipid A from *F. nucleatum* ATCC 51191 is reported in Figure 6. The spectrum showed in the *m/z* range 1346.7–1909.2, the presence of a heterogeneous pattern of signals relative to deprotonated [*M*−H]^−^ lipid A species that differed in the nature and number of fatty acid chains and in the phosphate content. Two main groups of signals at around *m/z* 1801.1 and 1881.1 were identified as hexa‐acylated lipid A species substituted by one or two phosphate groups, respectively (Table S1). In particular, as described below, the main peak at *m/z* 1881.1 matched with a bis‐phosphorylated lipid A carrying 14 : 0 (3‐OH) and 16 : 0 (3‐OH) as primary O‐ and N‐linked fatty acids, whereas two 14 : 0 residues corresponded to secondary acyl substituents. In addition, a cluster of peaks at about *m/z* 1670.9 and 1590.9 was assigned to bis‐ and mono‐phosphorylated penta‐acylated lipid A species lacking one 14 : 0 unit, whereas peaks at *m/z* 1444.7 and 1364.7 were attributed to bis‐ and mono‐phosphorylated tetra‐acylated lipid A species devoid of both one 14 : 0 and one 14 : 0 (3‐OH). Notably, the spectrum clearly showed differences of 28 amu (−CH_2_CH_2_‐ unit) diagnostic for the occurrence of lipid A species differing in the length of the acyl chains. A negative‐ion MS/MS analysis was conducted in order to establish the exact location of the acyl chains, as well as of the phosphate decorations of the mono‐phosphorylated lipid A species, with respect to the glucosamine disaccharide backbone. In particular, the MS/MS spectrum recorded on the precursor ion at *m/z* 1801.1 relative to a mono‐phosphorylated hexa‐acylated lipid A species, is reported in Figure S5.

**Figure 7:**
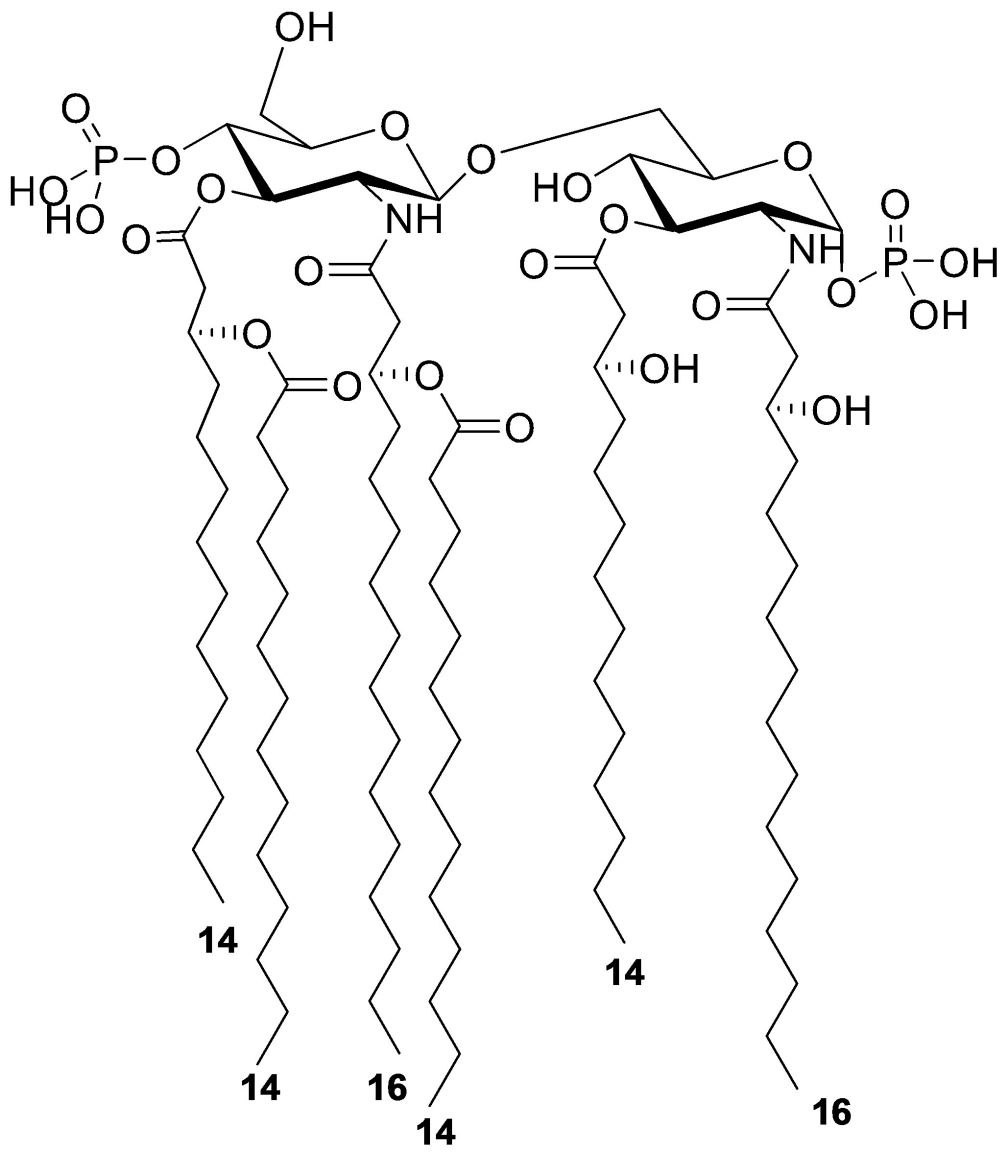
Proposed structure for the main bis‐phosphorylated hexa‐acylated lipid A species of *F. nucleatum* ATCC 51191. The absolute configuration of the primary acyl chains and the anomeric configuration of the two GlcN residues is based on literature data.

The spectrum clearly showed the presence of two main peaks matching with ions originated from the loss of one 14 : 0 (*m/z* 1573.0) and one 14 : 0 (3‐OH; *m/z* 1557.0) unit, respectively. An ion derived from the loss of a whole unit of a hydroxylated 14 : 0 bearing the secondary 14 : 0 substituent matched peak at *m/z* 1346.7; in contrast, an ion originated from the sequential loss of one 14 : 0 (3‐OH) and one 14 : 0 was attributed to peak at *m/z* 1328.7. Importantly, a peak that was crucial to define the location of the two secondary 14 : 0 acyl chains with respect to the glucosamine backbone was detected at *m/z* 738.2; this was attributed to an Y_1_‐type ion derived from the cleavage of the glycosidic linkage,^[26]^ which demonstrated that the phosphate group was on the reducing glucosamine that in turn was acylated by one 14 : 0 (3‐OH) and one 16 : 0 (3‐OH). In parallel, the presence of this fragmentation‐derived ion demonstrated that the secondary acyl substitutions occurred on the primary acyl chains of the sole non‐reducing glucosamine. Finally, the absence of any peak matching with the loss of a 16 : 0 (3‐OH) unit suggested that this fatty acid was present only as an acyl amide moiety. Therefore, by combining data from fatty acid compositional analysis and from MALDI MS and MS/MS, it was possible to establish the fine structure of the lipid A from *F. nucleatum* ATCC 51191 LPS whose main bis‐phosphorylated hexa‐acylated form is presented in Figure 7.

## Discussion

Using a combination of chemical, MS and NMR approaches, we determined the structure of the O‐antigen [→4)‐β‐d‐Glc*p*NAcA‐(1→4)‐β‐d‐Glc*p*NAc3NAlaA‐(1→3)‐α‐d‐Fuc*p*NAc4NR‐(1→], (R= Acetylated 60 %), and the heterogenous lipid A moiety of the LPS from *F. nucleatum* spp. *animalis* ATCC 51191. Among *F. nucleatum* strains there is a high variability of O‐antigen structures characterized to date.[^6^, ^13^, ^14^, ^15^, ^16^, ^17^, ^18^, ^19^] According to the Bacterial Carbohydrate Structure Database,[^25^, ^27^] *F. nucleatum* ATCC 51191 LPS trisaccharide represents a novel O‐antigen structure for bacterial LPS. The tool was used in the “composition” mode, by searching for a glycan containing the three units. This search produced no results, even though the queries with the single monosaccharides returned several hits: 63 results for β‐d‐Glc*p*NAcA, 132 for β‐d‐Glc*p*NAc3NAcA, and 167 for α‐d‐Fuc*p*NAc4NAc.

Importantly, α‐d‐Fuc*p*NAc4NAc was described for other strains of *F. nucleatum* such as MJR 7757 B^[14]^ and strain 10953.^[15]^ Such uncommon structural features of *F. nucleatum* ATCC 51191 O‐antigen raise questions on the immunomodulatory properties of this polysaccharide. Moreover, we showed that *F. nucleatum* ATCC 51191 lipid A consisted of a mixture of species with a certain degree of heterogeneity in both the acyl chains and phosphate content. In particular, the main species detected by negative‐ion MALDI‐TOF MS were identified as bis‐phosphorylated and hexa‐acylated with 14 : 0 (3‐OH) and 16 : 0 (3‐OH) as primary acyl chains, and two 14 : 0 as secondary fatty acids distributed in a 4+2 symmetry (Figure S5). This analysis was in agreement with the lipid A structure reported for *F. nucleatum* subsp. nucleatum strains,[^20^, ^28^] although no 12 : 0 fatty acid residue has been detected in *F. nucleatum* ATCC 51191 compared to data previously reported for *F. nucleatum* sp. *nucleatum* JCM 8532.^[29]^ It is worth noting that *F. nucleatum* ATCC 51191 lipid A is similar to that of *E. coli* in terms of acylation degree and distribution of the acyl chains (4+2); however, the length of the acyl chains (12 and 14 in *E. coli vs* 14 and 16 in *F. nucleatum* ATCC 51191 is different and may influence its immunopotency.^[10]^ Interestingly, the lipid A from the opportunistic pathogen *Burkholderia cenocepacia* shows the same acylation pattern as *F. nucleatum* ATCC 51191, although it only expresses tetra‐ and penta‐acylated lipid A species. Nevertheless, *B. cenocepacia* lipid A strongly activates human TLR4/MD‐2 signalling partly through the occurrence of the 16 : 0 (3‐OH) acyl chains.^[30]^ Therefore, the acylation profile of *F. nucleatum* ATCC 51191 lipid A and O‐antigen unique structure might significantly contribute to the immunostimulatory potency of this strain, and further studies will explore this aspect.

## Conclusion

*F. nucleatum* ssp. *animalis* ATCC 51191 produces an LPS whose repeating unit is a linear trisaccharide made up of [→4)‐β‐d‐Glc*p*NAcA‐(1→4)‐β‐d‐Glc*p*NAc3NAlaA‐(1→3)‐α‐d‐Fuc*p*NAc4NR‐(1→], with the N‐4 of the fucosamine partly acetylated (60 %). The lipid A shows a discrete heterogeneity with the main bis‐phosphorylated hexa‐acylated form carrying 14 : 0, 14 : 0 (3‐OH), and 16 : 0 (3‐OH) acyl chains organized in a 4+2 symmetry with the respect to the disaccharide backbone. This work provides novel structural insights into *F. nucleatum* LPS strain‐specific features boosting the interest in elucidating the molecular details of the interaction between *F. nucleatum* LPS and the host immune receptors.

## Experimental Section

***F. nucleatum*** **growth and LPS purification**: *F. nucleatum* ssp. *animalis* ATCC 51191 cells were grown anaerobically (85 % N_2_, 5 % CO_2_, 10 % H_2_) at 37 °C in tryptic soy broth medium (Becton Dickinson) supplemented with 5 μg/mL hemin (Sigma–Aldrich) and 1 μg/mL menadione (Sigma‐Aldrich). The bacteria from a culture of 8 L were harvested at OD_600 nm_ of 0.7–0.9^[31]^ following centrifugation at 9000 *g* for 15 min. The cell pellet was freeze‐dried and the lyophilized cells (3 g) were extracted by the hot phenol/water method.^[21]^ Each phase was dialysed against distilled water to remove the phenol and then freeze‐dried. Afterwards, each dried phase was analysed by 12 % SDS‐PAGE and visualized by silver nitrate staining.^[32]^ LPS was found in the water phase of the water/phenol extraction (yield 76.7 mg/g of cells). This phase was further purified by enzymatic digestion using DNAse, RNAse and proteinase K (yield 23.0 %),^[33]^ followed by centrifugation at 6000 rpm, for 30 min at 4 °C and ultracentrifugation at 30 000 rpm *please give g value or rotor type* for 4 h at 4 °C.

**Lipid A and O‐antigen isolation**: *F. nucleatum* ATCC 51191 LPS (12 mg) was subjected to mild acid hydrolysis (acetic acid 1 %, 100 °C, 2 h), yielding the lipid A fraction (1 mg) and a water‐soluble fraction (9 mg). Lipid A was separated by centrifugation (3000 rpm, RT, 15 min), and the polysaccharide part present in the supernatant was further purified by size exclusion chromatography (78.6 mL volume, 78.6 mL/h flow, NH_4_HCO_3_ 50 mM) using a Sephacryl S200 column. The eluate was monitored by a refractive index detector (Knauer GmbH – WellChrom Differential Refractometer K‐2301) and fractions were pooled accordingly. The yield of the O‐antigen was of 0.41 mg per mg of LPS.

**Compositional analysis**: The monosaccharide content was determined following the acetylated *O*‐methyl glycoside derivatives method.^[33]^ Briefly, *F. nucleatum* ATCC 51191 LPS (0.5 mg) was treated with 1.25 M HCl/MeOH at 80 °C for 16 h followed by an acetylation step with acetic anhydride (50 μL) in pyridine (100 μL) at 80 °C for 30 min. The fatty acid composition was determined by analysing the hexane extract of the sample after methanolysis as previously reported.^[33]^

All chemical derivatives were analysed by using a gas‐liquid chromatography (GLC‐MS) Agilent Technologies 7820 A (Santa Clara, CA, USA) equipped with a mass selective detector 5977B and a HP‐5 ms capillary column Agilent, Italy (30 m×0.25 mm i.d., 0,25 μm as film thickness, flow rate 1 mL/min, He as carrier gas). Electron impact mass spectra were recorded with ionization energy of 70 eV and an ionizing current of 0.2 mA. The temperature program used was: 150 °C for 5 min, 150 up to 300 °C at 10 °C/min, 300 °C for 12 min.

**NMR spectroscopy**: For structural assignments of the O‐antigen, ^1^H NMR and 2D NMR spectra were recorded using a Bruker 600 MHz spectrometer equipped with a reverse cryo‐probe with gradients along the *z* axis. The sample was solved at a concentration of 1 mg in 550 μL of D_2_O and the spectra were calibrated with internal acetone (*δ*_H_=2.225 ppm; *δ*_C_=31.45 ppm). In addition, for the detailed O‐antigen analysis, the spectra were recorded at different conditions as follows: in alkaline conditions by adding 4 μL of NaOD 4 M to 550 μL of D_2_O, 25 °C; in acid conditions by adding 4 μL of DCl conc. to 550 μL of D_2_O, 37 °C. The variation of the chemical shifts as a result of the different pH employed, eliminated some signals overlap, thus facilitating the structural characterization.

Total correlation spectroscopy (TOCSY) experiments were performed with spinlock times of 100 ms using data sets (t1×t2) of 2048×512 points. Nuclear Overhauser enhancement spectroscopy (NOESY) experiments were performed using data sets (t1×t2) of 2048×512 points with mixing times of 200 ms. Heteronuclear single‐quantum coherence (HSQC), and heteronuclear multiple‐bond correlation (HMBC) experiments were performed in the ^1^H‐detection mode by single‐quantum coherence with proton decoupling in the ^13^C domain using data sets of 2048×512 points. HSQC was performed using sensitivity improvement and the phase‐sensitive mode using echo/antiecho gradient selection, with multiplicity editing during the selection step.^[34]^ HMBC was optimized on long‐range coupling constants, with a low‐pass *J* filter to suppress one‐bond correlations, using gradient pulses for selection. Moreover, a 60 ms delay was used for the evolution of long‐range correlations. HMBC spectra were optimized for 6–15 Hz coupling constants. The data matrix in all the heteronuclear experiments was extended to 4092×2048 points and transformed by applying a qsine or a sine window function.^[35]^

**MALDI‐TOF MS and MS/MS**: MALDI‐TOF MS and MS/MS analysis were performed on an ABSCIEX TOF/TOF^TM^ 5800 Applied Biosystems mass spectrometer equipped with a Nd : YLF laser with a λ of 345 nm, a b500‐ps pulse length and a repetition rate of up to 1000 Hz. The lipid A was dissolved in CHCl_3_/MeOH (1 : 1, *v*/*v*), as previously described.[^36^, ^37^] The matrix was the trihydroxyacetophenone (THAP) dissolved in CH_3_OH/0.1 % TFA/CH_3_CN (7 : 2 : 1, *v*/*v*/*v*) at a concentration of 75 mg/mL. The lipid A solution (0.5 μL) and the matrix solution (0.5 μL) were deposited on the MALDI plate and dried at room temperature. All spectra were a result of the accumulation of 2000 laser shots, whereas 6000–7000 shots were summed for the MS/MS data acquisitions as described previously.^[38]^ Each experiment was performed in triplicate.

## Conflict of interest

The authors declare no conflict of interest.

## Supporting information

As a service to our authors and readers, this journal provides supporting information supplied by the authors. Such materials are peer reviewed and may be re‐organized for online delivery, but are not copy‐edited or typeset. Technical support issues arising from supporting information (other than missing files) should be addressed to the authors.

Supplementary
