# Supplementary material for: Structure of the O‐Antigen and the Lipid A from the Lipopolysaccharide of Fusobacterium nucleatum ATCC 51191
Source: Chembiochem. 2020 Dec 14;22(7):1252–60. doi: 10.1002/cbic.202000751 (PMC8048906; doi:10.1002/cbic.202000751)
Supplement: Supplementary file 1 — Supplementary [file CBIC-22-1252-s001.pdf]

ChemBioChem

Supporting Information

Structure of the O-Antigen and the Lipid A from the Lipopolysaccharide of *Fusobacterium nucleatum* ATCC 51191

Pilar Garcia-Vello, Flaviana Di Lorenzo, Dimitra Lamprinaki, Anna Notaro, Immacolata Speciale, Antonio Molinaro, Nathalie Juge, and Cristina De Castro*

Author Contributions

P.G.-V. Formal analysis:Lead; Investigation:Lead; Writing – original draft:Lead
F.D. Investigation:Equal; Writing – original draft:Equal
D.L. Resources:Supporting
A.N. Investigation:Supporting
I.S. Investigation:Supporting
A.M. Conceptualization:Supporting; Investigation:Supporting; Supervision:Supporting; Writing – review & editing:-Supporting
N.J. Resources:Supporting; Writing – review & editing:Supporting
C.D. Conceptualization:Lead; Investigation:Lead; Supervision:Lead; Writing – review & editing:Lead

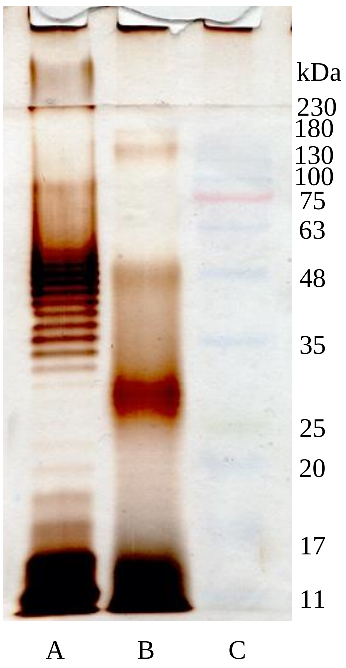

Figure S1. Electrophoresis analysis of bacterial LPS. **A.** *E. coli* O111:B4 LPS (8 μg). **B.** *F. nucleatum* ATCC 51191 LPS (8 μg) **C.** BlueEye protein standard. The samples were run on a 12 % SDS-PAGE and visualized by silver staining.

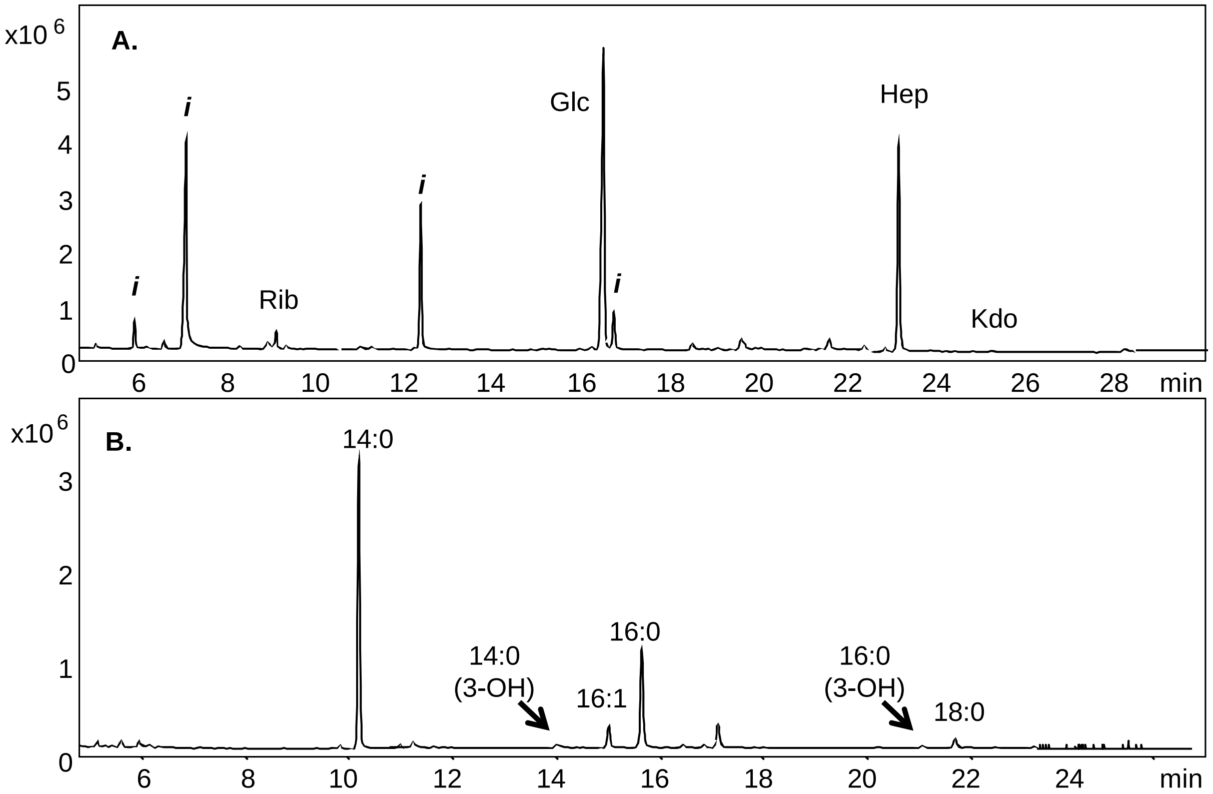

Figure S2. GC-MS profile of *F. nucleatum* ATCC 51191 LPS **A**. acetylated methyl glycosides, **B.** lipid compositional analysis. I: impurities; 16:1 and 18:0 are impurities or cell derived fatty acids.

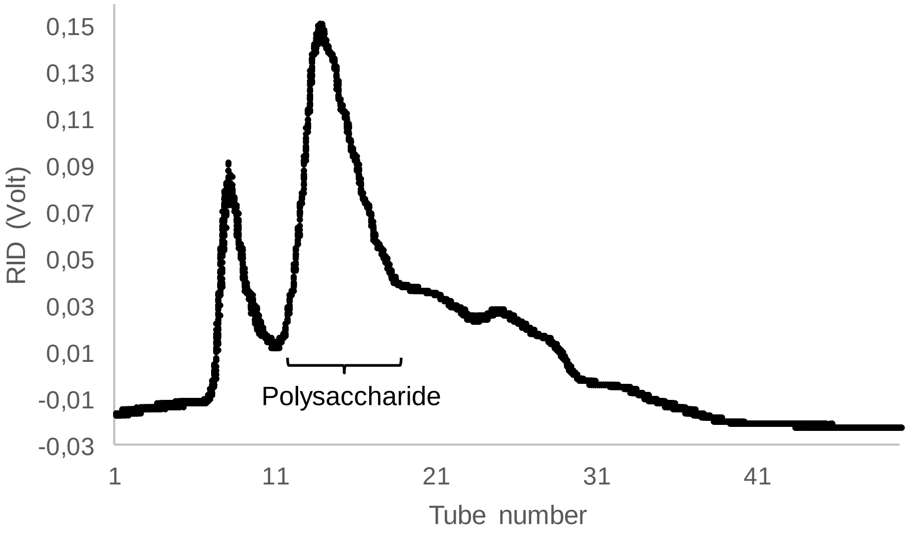

Figure S3: Chromatogram profile of *F. nucleatum* ATCC 51191 O-antigen purification on a Sephacryl S200 column. The O-antigen corresponds to the second (highest) peak of the chromatogram.

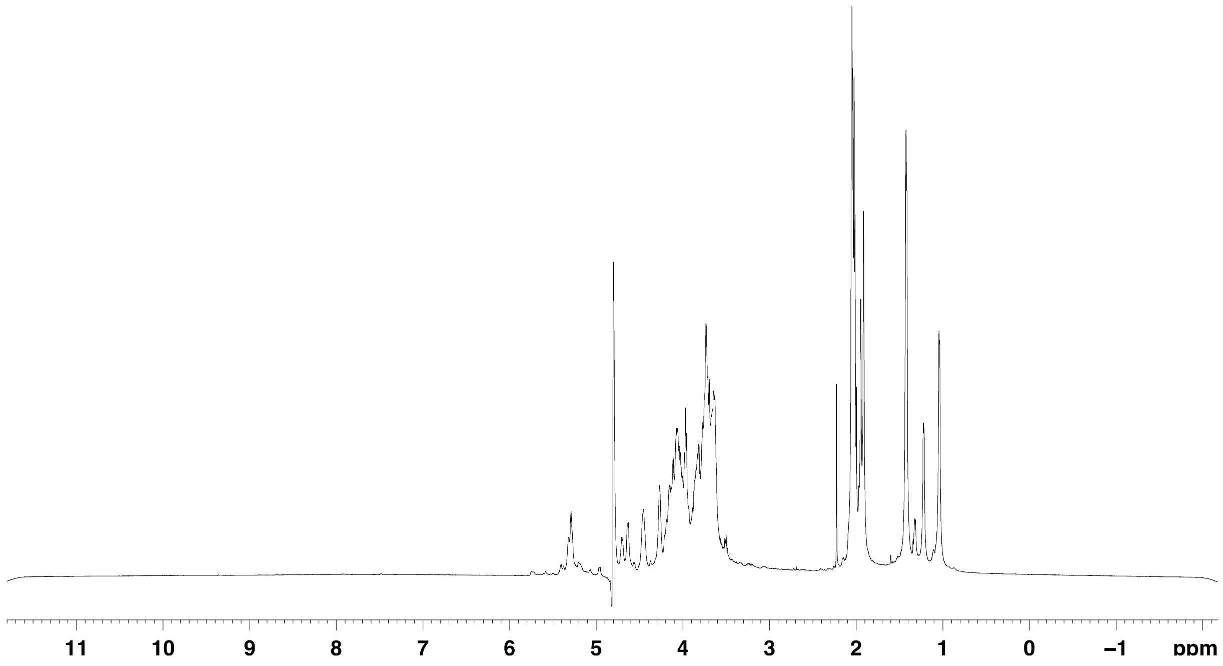

Figure S4: ^{1}H NMR spectrum of the O-antigen of *F. nucleatum* ATCC 51191 showing the lack of signals around 8 ppm and therefore the absence of a formyl group (600 MHz, 25 °C, 550 μL of D_2O, neutral pH).

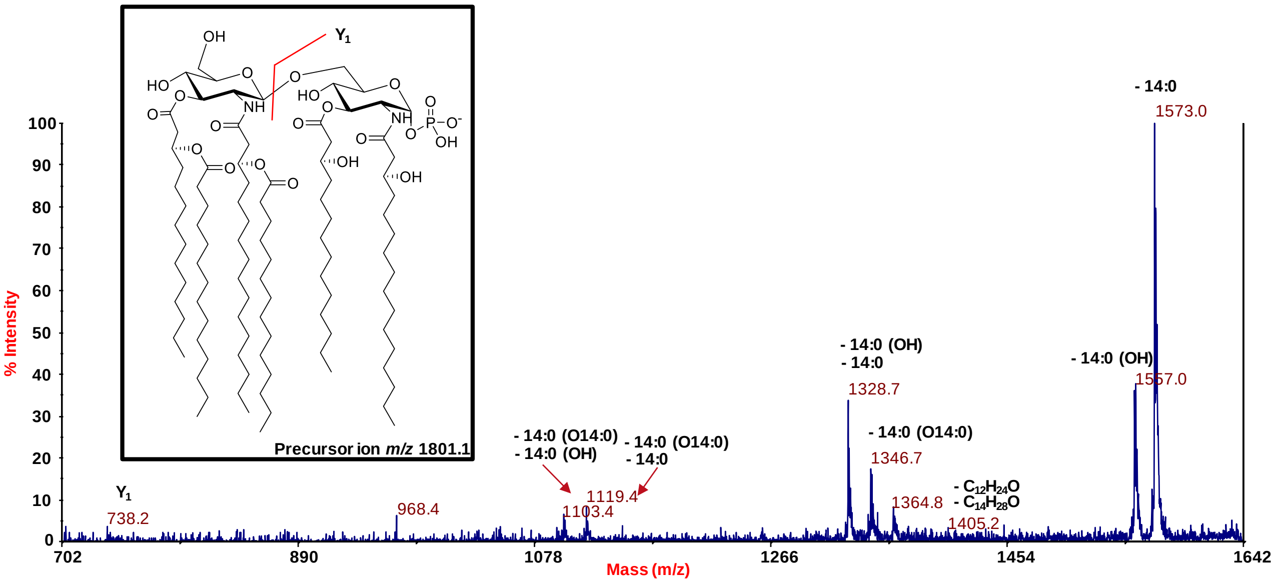

Figure S5. Negative-ion MALDI MS/MS spectrum of precursor ion at *m/z* 1801.1 of the lipid A of *F. nucleatum* ATCC 51191. This is a representative ion peak of the cluster ascribed to hexa-acylated lipid A species decorated by two phosphates. The main fragments' assignment is indicated in the spectrum. The proposed structure is reported in the inset. The loss of $C_{12}H_{24}O$ (184 mass units) and $C_{14}H_{28}O$ (212 mass units) is also indicated and was due to a rearrangement typically occurring on primary acyl chains only when their 3-OH group is free, thus contributing to the establishment of the location of the secondary acyl substitution.

Table S1. The main MALDI-TOF MS ion peaks *F. nucleatum* ATCC 51191 lipid A. The table reports the predicted mass and the proposed interpretation of the substituting fatty acids and phosphates on the *F. nucleatum* ATCC 51191 lipid A backbone. See Figure 6 for full spectrum.

Predicted mass (Da)	Observed ion peaks (*m/z*)	Acyl substitution	Proposed fatty acid/phosphate composition
1364.96	1364.68	Tetra-acyl	$HexN_2P$ [14:0(3-OH)] $[16:0(3\text{-}OH)]_2$ (14:0)
1444.92	1444.69	Tetra-acyl	$HexN_2P_2$ [14:0(3-OH)] $[16:0(3\text{-}OH)]_2$ (14:0)
1591.15	1590.86	Penta-acyl	$HexN_2P$ $[14:0(3\text{-}OH)]_2$ $[16:0(3\text{-}OH)]_2$ (14:0)
1671.11	1670.87	Penta-acyl	$HexN_2P_2$ $[14:0(3\text{-}OH)]_2$ $[16:0(3\text{-}OH)]_2$ (14:0)
1801.35	1801.15	Hexa-acyl	$HexN_2P$ $[14:0(3\text{-}OH)]_2$ $[16:0(3\text{-}OH)]_2$ $(14:0)_2$
1881.31	1881.15	Hexa-acyl	$HexN_2P_2$ $[14:0(3\text{-}OH)]_2$ $[16:0(3\text{-}OH)]_2$ $(14:0)_2$
